# A rare case of permanent pacemaker-induced takotsubo cardiomyopathy in a male patient

**DOI:** 10.5339/qmj.2022.4

**Published:** 2022-02-22

**Authors:** Imran Moinudddin, Ibrar Anjum, Umer Zia, Muhammad Bila Zia, Sajid Ali

**Affiliations:** ^1^Baylor St Luke's Medical Center, Houston, Texas, United States; ^2^Vassar Brother Medical Center, NY, United States E-mail: ibrar.anjum@gmail.com; ^3^Houston Cardiology Consultants, Texas, United States

**Keywords:** Takotsubo cardiomyopathy, pacemaker implantation, heart block, pacemaker, broken heart syndrome

## Abstract

Takotsubo cardiomyopathy is a type of non-ischemic cardiomyopathy that usually appears after a stressful event or in a woman and is rarely seen after pacemaker implantation (PMI). Herein, we present the case of a 65-year-old man with PMI because of a 2:1 atrioventricular nodal block who had a syncopal episode later in the day of the procedure. Echocardiography showed septal and apical hypokinesis with reduced ejection fraction suggestive of takotsubo cardiomyopathy. Before PMI, echocardiography showed normal left ventricular function with no wall-motion abnormality. Coronary angiography showed no coronary artery stenosis. The patient was seen again in the clinic 1 month later, and repeat echocardiography showed improvement of ejection fraction to 55% with no wall-motion abnormality. Generally, the complication rate after PMI is very low and includes infection, hematoma, lead dislocation, or allergic reaction at the site. The clinicians must be aware of potentially rare complications that can occur after PMI, such as takotsubo cardiomyopathy.

## Introduction

Takotsubo cardiomyopathy (TCM), also known as broken heart syndrome, typically occurs following a stressful emotional event, especially in women. It is characterized by transient apical myocardial dysfunction in the absence of obstructive coronary artery disease. On **echocardiography,** the affected heart resembles a Japanese octopus trap **(tako-tsubo)**; thus, it is called TCM ^1,2^. This syndrome is frequently misdiagnosed as an acute coronary syndrome related to occlusive epicardial coronary artery disease; however, coronary angiography is generally normal. Despite its frequently dramatic clinical presentation, patients usually recover their left ventricular (LV) systolic function from days to weeks. The underlying mechanism has not been established; however, major hypotheses to its etiology include microvascular dysfunction with **endogenous and exogenous** catecholamine-induced cardiomyopathy ^3^. Many other conditions have been reported, which can induce TCM in the absence of a stressful event, including snake bites, pancreatitis, postoperative-induced cases, and medication-induced (capecitabine) cases. Herein, we present a unique case of TCM in a 65-year-old man who underwent permanent pacemaker placement.

## Case Presentation

**Figure 1.**
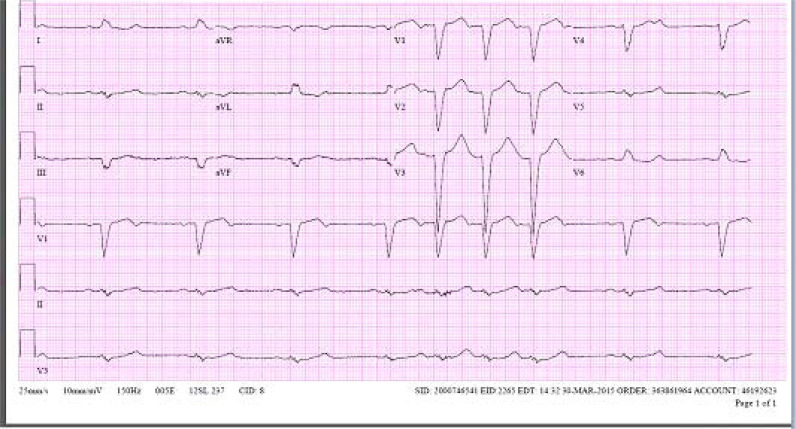
Electrocardiography showed a 2:1 atrioventricular (AV) nodal heart block and left bundle branch block (LBBB).

**Figure 2.**
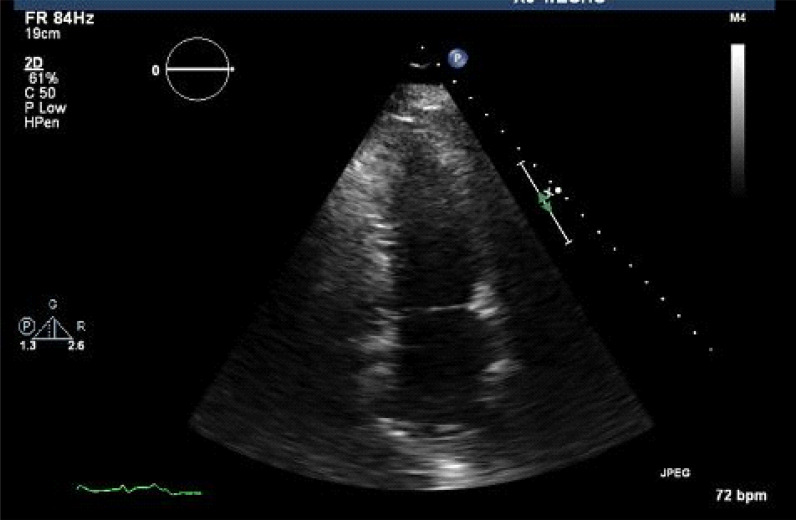
Echocardiography showed normal left ventricular function with no wall-motion abnormality.

A 65-year-old white man with a history of type 2 diabetes mellitus and hypertension on metoprolol presented to the emergency department with diarrhea and generalized weakness for 3 days. On vitals, he was hemodynamically stable. Physical examination was unremarkable. The electrocardiogram (ECG) showed a new 2:1 atrioventricular nodal block (AVN) and old left bundle branch block (LBBB) (Figure 1). Metoprolol was immediately discontinued. Echocardiography, which was performed 1 day after admission, showed normal LV function with no wall-motion abnormality (Figure 2). On persantine nuclear stress test, there was a hibernation of the inferior wall of the LV myocardium. The patient subsequently underwent left heart catheterization, which showed normal coronary arteries and normal ventricular function.

After 48 h, the patient remained in 2:1 AVN block, and we decided on the placement of a permanent pacemaker. On day 4 after admission, a dual-chamber pacemaker was successfully implanted. After the procedure, the device was checked and functioning appropriately. A few hours after the procedure, a nurse noticed a syncopal episode. On vitals, the patient was hypotensive, which required immediate workup.

Chest X-ray findings were normal with no evidence of pneumothorax. Repeat ECG showed sinus rhythm, with ventricular pacing **without acute ST-segment changes**.

**Figure 3.**
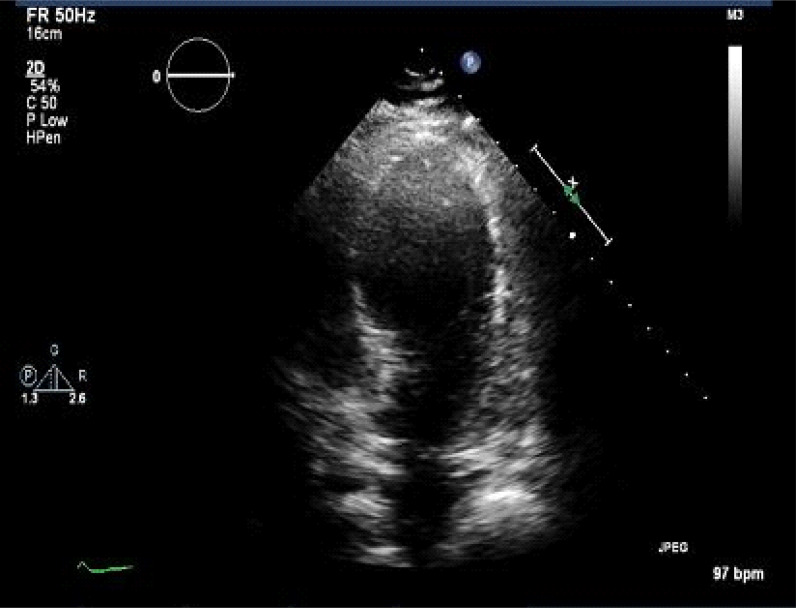
Echocardiography showed septal, lateral mid-chamber, and apical hypokinesis with dilatation.

Troponin was mildly elevated to 0.04 μg/L (reference range <  0.03 μg/L). Repeat transthoracic echocardiography showed acute findings of septal, lateral mid-chamber, and apical hypokinesis with dilatation suggestive of TCM. Pericardial effusion was not detected (Figure 3). At this time, the ejection fraction significantly decreased to 35%. The patient was treated with conventional heart failure therapy, including angiotensin-converting enzyme inhibitors and beta-blockers. He was discharged from the hospital and remained asymptomatic. The patient was seen 1 month after for follow-up in the cardiology clinic, and he remained free from symptoms. His pacemaker was rechecked and was functioning properly. At this time, a repeat transthoracic echocardiography showed an ejection fraction of 55%, no wall-motion abnormalities, and grade one diastolic dysfunction.

## Discussion

The presented case is unique, as this is an acute episode of TCM diagnosed after permanent pacemaker placement. The Mayo Clinic defines TCM by four defining characteristics: transient **hypokinesis, akinesis**, or dyskinesis of the LV mid-segments (more than one coronary territory) with or without apical involvement with a stressful trigger present in the absence of obstructive coronary disease, new ECG abnormalities (either ST-segment elevation and/or T-wave inversion), or modest elevation in cardiac troponin level with the absence of pheochromocytoma or myocarditis ^4^.

In our patient, ECG changes were precluded, as he was ventricularly paced and had underlying LBBB. He did have a mild troponin elevation of 0.04, which is typical for TCM. He did not have clinical manifestations of pheochromocytoma and thus did not warrant a workup for it. Echocardiography showed the characteristic mid- to apical ballooning with severe hypokinesis of the apex to the mid-LV cavity. In our patient, the pacemaker implant was the only identified precursor that led to the diagnostic findings. We also excluded coronary artery disease; thus, the patient met the Mayo Clinic criteria for the diagnosis of TCM.

Eleven cases of TCM have been reported in a narrative literature review and nearly exclusively affect older women. There was only one case report of an elderly male patient who had a permanent pacemaker that resulted in TCM but had a cardiogenic shock and was administered isoproterenol, which was suspected to be the cause of his TCM. No case report in our literature search has shown permanent pacemaker placement as the sole cause of TCM in a male patient.

Studies have shown that chronic right ventricular (RV) pacing causes abnormal electrical and mechanical activation patterns of the ventricles, resulting in mechanical dyssynchrony and deterioration of the LV function, but this does not indicate how acute RV pacing could result in TCM ^5–7^. A study was conducted on 25 individuals testing the effect of RV pacing on the LV function, and none of them showed any evidence of structural heart disease using two-dimensional speckle tracking strain imaging. This study showed that direct stimulation of RV induces an abnormal activation sequence, resulting in asynchronous ventricular contraction; however, this asynchrony would not account for the multi-vessel territory involvement seen in TCM ^8^. Another study in an animal model showed that rapid RV pacing required compensatory reductions in myocardial contractility to balance the myocardial metabolic requirements causing acute myocardial hibernation ^9^. Again, this is precluded in our case, as our patient never had rapid pacing.

Post RV pacemaker placement with chronic pacing at a threshold of 40% pacing burden is a known potential cause of LV cardiomyopathy, with an incidence of up to 19%. Additionally, men with wider native QRS duration (particularly >115 ms) are at increased risk of pacing-induced cardiomyopathy ^10^. However, the incidence of acute transient myocardial dysfunction is unknown, and in truth, only a few cases have been reported. We present this case to recognize that permanent pacemaker implantation may be a potential cause of TCM. RV pacing can cause deleterious effects, and this should be kept in mind as a potential post pacemaker implantation outcome. Further, the mechanism of acute myocardial dysfunction needs to be evaluated to see if a correlation can be found between chronic and acute pacemaker-induced cardiomyopathy.

## Conclusion

The rate of complications after pacemaker implantation is very low and includes infection, **hematoma, lead dislocation, perforation, pneumothorax, or allergic reaction at the site.** TCM usually occurs in women after stressful events and is rarely seen as an adverse event in a man after invasive procedure for pacemaker implantation. Clinicians must be aware of potentially rare complications that can occur after pacemaker implantation, such as TCM.

